# Genetic diversity and population structure of Anatolian Hair goats, an ancient breed

**DOI:** 10.5194/aab-67-13-2024

**Published:** 2024-01-11

**Authors:** Aylin Demiray, Zühal Gündüz, Nezih Ata, Onur Yılmaz, İbrahim Cemal, Aynur Konyalı, Zeynep Semen, Arif Altuntaş, Ali Atik, Ahmet Akçay, Hüseyin Baş, Hasan Hüseyin Şenyüz

**Affiliations:** 1 Department of Breeding and Genetics, International Center for Livestock Research and Training, Ankara, Türkiye; 2 Department of Agricultural Biotechnology, Aydın Adnan Menderes University, Aydın, Türkiye; 3 Department of Animal Science, Aydın Adnan Menderes University, Aydın, Türkiye; 4 Department of Animal Science, Çanakkale Onsekiz Mart University, Çanakkale, Türkiye; 5 Department of Biochemistry, Dokuz Eylül University, İzmir, Türkiye; 6 Department of Biochemistry, Ankara University, Ankara, Türkiye; 7 Department of Livestock Research, Bahri Daǧdaş International Agricultural Research Institute, Konya, Türkiye; 8 Central Research Laboratory Application and Research Center, Mardin Artuklu University, Mardin, Türkiye; 9 Department of Animal Nutrition and Nutritional Disease, Necmettin Erbakan University, Konya, Türkiye

## Abstract

This study aimed to investigate the genetic characterization and diversity of Hair goats from 10 regions using 20 microsatellite markers. A total of 522 alleles were observed. The INRA0023 locus exhibited the greatest number of alleles (48), while the DRBP1 locus had the highest effective allele number (16.27), and the BM1818 and DRBP1 loci had the highest polymorphic information content value (0.94). The expected heterozygosity value ranged from 0.85 (ILSTS011) to 0.94 (BM1818, SRCRSP15, and DRBP1). The Hair goat populations in Konya and Hatay displayed the lowest and highest allele numbers, with values of 10.40 and 16.25, respectively. The fixation index ( $F_{\mathrm{IS}}$ ) values are significant in defining population structures and determining the extent of heterozygosity losses. The $F_{\mathrm{IS}}$ values exhibited a range of 0.031 in Muǧla to 0.226 in Burdur. A total of 107 unique alleles were identified in Hair goat populations. However, it is noteworthy that, out of all the alleles, only 25 had a frequency exceeding 5 %. The results indicate that the microsatellite markers utilized demonstrate sufficient levels of polymorphism, making them appropriate for efficiently investigating the genetic variability of Hair goat populations.

## 1 Introduction

Goats are regarded to be a crucial component of animal production systems in Türkiye, particularly in rural regions where they are reared in extensive conditions. Goat breeding is typically conducted in regions where agricultural cultivation and other forms of animal husbandry are not viable, particularly in the inner-forest areas. Hair goats, which constitute 97.8 % of Türkiye's goat population and for which many studies have been carried out to define their productivity characteristics, are mostly raised in the mountainous regions of Türkiye (Simsek et al., 2007; Atay et al., 2010; Cemal et al., 2019; Sirin, 2019; TUIK, 2022; Cemal et al., 2021). Although Hair goats are bred in all regions of Türkiye, they are known for their resilience to severe climatic conditions. The Hair goat is a breed that exhibits disease resistance due to its sturdy physique, which is well suited to the challenging climate and terrain of Anatolia (Erduran and Yaman, 2012; Koluman et al., 2013; Keskin et al., 2017; Daskiran et al., 2018).

Breeding programs aimed at improving productivity in farm animals have been observed to potentially compromise their adaptive capabilities. Nevertheless, these programs also emphasize the critical importance of the conservation of genetic resources (Karaca and Cemal, 1998). Research on the conservation of genetic resources is becoming increasingly significant, and investigations in this field are expanding globally, including in Türkiye. The initial stage in preserving diversity among domestic animals is to possess a thorough understanding of their genetic variability. It is of utmost importance to accurately determine the breed that requires protection for the purpose of conservation in order to prevent the loss of valuable allele combinations present in the gene pool. Numerous studies have been conducted on the genetic diversity of Hair goats in Türkiye (Bulut et al., 2016; Gurler and Bozkaya, 2013; Karsli et al., 2020; Tefiel et al., 2020; Gul et al., 2020; Karsli et al., 2022). However, this study was carried out in a wide geographical area where Hair goats are grown intensively, using a relatively high number of microsatellite markers compared to other studies.

It can be asserted that there exists a significant phenotypic variation among the populations of Hair goats that are raised in nearly all regions of Türkiye (Anonymous, 2023). Most of the goat breeding in Türkiye is carried out under extensive conditions. Within the scope of the National Genetic Improvement Project for Small Ruminants at Breeders' Conditions, which was launched by the General Directorate of Agricultural Research in 2005, breeding plans were carried out for animal species and breeds, and modern breeding organizations were put into action under field conditions. Hair goats have also been included in breeding organizations based on the open-core breeding model in many provinces of Türkiye. Records such as productivity and pedigree, which are needed to estimate genetic parameters for the breed, have begun to be obtained (Karaca et al., 2014).

In the past century, statistical techniques have been extensively utilized in breeding research, particularly in the field of livestock (Beuzen et al., 2000). Advancements in molecular biology and statistical techniques have facilitated the identification and utilization of significant quantitative trait loci (QTL) and genomic variations for enhancing the genetics of farm animals (Montaldo and Meza-Herrera, 1998).

The determination of population variation and genetic diversity in farm animals is of great significance in uncovering population structures and inter-population relationships and in devising national breeding and conservation programs. It is widely recognized that indigenous breeds are progressively being supplanted by high-yielding breeds, resulting in the potential extinction of certain breeds without proper identification. The current investigation aimed to ascertain the genetic variability and population structure of Hair goats, a breed commonly reared in Türkiye.

## 2 Material and methods

### 2.1 Sampling and DNA isolation

**Figure 1:**
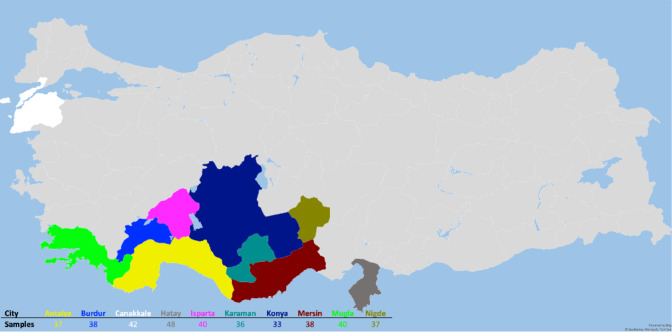
Sampling locations.

The animal material for the study consisted of 389 Hair goats, which were non-consanguineous to each other and were raised in 10 different provinces (Çanakkale, Karaman, Burdur, Niǧde, Muǧla, Mersin, Konya, Antalya, Isparta, and Hatay) in Türkiye. The animals included in the study are part of the breeding program. In this context, the selection of animals that constitute the animal population for the study was based on pedigree records. According to FAO (2011), genetic diversity studies conducted with microsatellites, one of the genetic markers, should involve a minimum of 25 unrelated individuals. In the presented study, sample numbers for animal material were selected according to this criterion. The figure depicting the sampling locations is presented in Fig. 1.

Blood was collected from the jugular vein of the animals using a needle and a vacuum tube containing K3EDTA. The collected blood was stored at - 20 ${}^{\circ }$ C until molecular genetic analysis was conducted. The genomic DNA was isolated from the blood samples using the salt precipitation technique, as previously outlined by Miller et al. (1988) and Montgomery and Sise (1990). After the isolation of DNA, its quantity and quality were evaluated using the NanoDrop 2000 spectrophotometer, which was produced by Thermo Scientific, USA.

### 2.2 PCR amplification and fragment analysis

In this study, 20 microsatellite markers were used, following the recommendation of the Food and Agriculture Organization (FAO, 2011). Loci that are likely to overlap due to their allelic ranges are marked with different fluorescent dyes. Loci without the possibility of overlap were marked with the same fluorescent dye, while those with the potential for overlap were marked with a different fluorescent dye. For this reason, forward primers of microsatellites were labeled with WellRed dye (D4, D3, or D2), which is compatible with the genetic analyzer (Beckman Coulter GeXP) used in the study to facilitate capillary electrophoresis and fragment analysis. Table 1 provides a comprehensive summary of the multiplex clusters generated by microsatellites, including the fluorescent dyes employed for labeling.

During the PCR stage, 50 ng of genomic DNA and sterile distilled water were used to amplify specific regions of the primer. The PCR tube contained a total volume of 20 µ L, which included 1X PCR buffer, 2.0 mM MgCl2, 0.20 mM dNTP mixture (dATP, dTTP, dGTP, dCTP), 0.10 µ M of both forward and reverse primers, and 1 U of Taq DNA Polymerase (ABM-G009). The touchdown PCR method, as described by Hecker and Roux (1996), was used for DNA amplification (Table 2). The PCR fragments labeled with fluorescence were separated by means of capillary electrophoresis, utilizing a Beckman Coulter GeXP Genetic Analyzer (Beckman Coulter, Inc., USA).

**Table 1:** Summary information of microsatellites used for analysis in the study.

Multiplex	Dye	Microsatellite	Primer sequences		Allelic range
M1	D3	OarFCB20	F	AATGTGTTTAAGATTCCATACAGTG	93–112
			R	GGAAAACCCCCATATATACCTATAC	
	D3	INRA0005	F	CAATCTGCATGAAGTATAAATAT	135–149
			R	CTTCAGGCATACCCTACACC	
	D3	INRA0023	F	GAGTAGAGCTACAAGATAAACTTC	195–225
			R	TAACTACAGGGTGTTAGATGAACTC	
	D3	ILSTS011	F	GCTTGCTACATGGAAAGTGC	250–300
			R	CTAAAATGCAGAGCCCTACC	
	D4	SRCRSP9	F	AGAGGATCTGGAAATGGAATC	99–135
			R	GCACTCTTTTCAGCCCTAATG	
	D4	SRCRSP15	F	CTTTACTTCTGACATGGTATTTCC	172–198
			R	TGCCACTCAATTTAGCAAGC	
	D4	TCRVB6	F	GAGTCCTCAGCAAGCAGGTC	217–255
			R	CCAGGAATTGGATCACACCT	
	D2	OarAE54	F	TACTAAAGAAACATGAAGCTCCCA	115–138
			R	GGAAACATTTATTCTTATTCCTCAGTG	
	D2	BM1818	F	AGCTGGGAATATAACCAAAGG	248–278
			R	AGTGCTTTCAAGGTCCATGC	
M2	D3	McM0527	F	GTCCATTGCCTCAAATCAATTC	165–187
			R	AAACCACTTGACTACTCCCCAA	
	D3	CSRD0247	F	GGACTTGCCAGAACTCTGCAAT	220–247
			R	CACTGTGGTTTGTATTAGTCAGG	
	D3	INRABERN185	F	CAATCTTGCTCCCACTATGC	261–289
			R	CTCCTAAAACACTCCCACACTA	
	D4	SRCRSP0023	F	TGAACGGGTAAAGATGTG	85–123
			R	TGTTTTTAATGGCTGAGTAG	
	D4	ILSTS0087	F	AGCAGACATGATGACTCAGC	137–155
			R	CTGCCTCTTTTCTTGAGAG	
	D4	SRCRSP0005	F	GGACTCTACCAACTGAGCTACAAG	158–180
			R	TGAAATGAAGCTAAAGCAATGC	
	D4	DRBP1	F	ATGGTGCAGCAGCAAGGTGAGCA	195–229
			R	GGGACTCAGTCTCTCTATCTCTTTG	
	D4	INRABERN172	F	CCACTTCCCTGTATCCTCCT	234–256
			R	GGTGCTCCCATTGTGTAGAC	
	D4	HSC (OLADRB)	F	CTGCCAATGCAGAGACACAAGA	267–301
			R	GTCTGTCTCCTGTCTTGTCATC	
	D2	SRCRSP3	F	CGGGGATCTGTTCTATGAAC	98–122
			R	TGATTAGCTGGCTGAATGTCC	
	D2	BM1329	F	TTGTTTAGGCAAGTCCAAAGTC	160–182
			R	AACACCGCAGCTTCATCC	

### 2.3 Statistical analysis

Various genetic parameters were computed to assess the genetic diversity of the population. These parameters included the number of alleles (Na), mean number of alleles (MNa), effective number of alleles (Ne), polymorphic information content (PIC), observed heterozygosity (Ho), expected heterozygosity (He), and Wright's F statistics ( $F_{\mathrm{IT}}$ , $F_{\mathrm{IS}}$ , $F_{\mathrm{ST}}$ ) (Wright, 1990; Weir and Cockerham, 1984). Additionally, compliance with the Hardy–Weinberg equilibrium was evaluated. The software programs GenAlEx (Peakall and Smouse, 2005, 2012), POPGENE (Yeh et al., 1997), and CERVUS 3.0.3 (Marshall, 1998/2006; Kalinowski et al., 2007) were utilized for these calculations. The software applications Populations 1.2.32 (Langella, 1999) and FigTree 1.4.2 (Rambout, 2006) were employed to generate a neighbor-joining (NJ) phylogenetic tree using Nei's Da distance matrix (Nei et al., 1983) for the populations being investigated. The reliability of the dendrogram's structure was evaluated by means of bootstrap resampling ( n=1000 ). The diversity between breeds ( $D_{\mathrm{ST}}$ ), coefficient of gene differentiation ( $G_{\mathrm{ST}}$ ), and Nei's gene diversity ( $H_{T}$ ) were computed using the FSTAT 2.9.3 program (Goudet, 2001). The present study utilized the AFC sur populations module of the GENETIX v4.05 software (Belkhir et al., 2001) to perform factorial correspondence analysis. This analysis aimed to investigate any possible admixtures that may have taken place among the populations under study. The Bayesian approach was employed to examine the population structures via cluster analysis techniques, utilizing the STRUCTURE software (Hubisz et al., 2009; Falush et al., 2003, 2007; Pritchard et al., 2000). The STRUCTURE analysis employed an admixture model and independent allele frequencies, with a length value of 20 000 being set. The number of iterations for the Markov-chain–Monte-Carlo was set to 100 000, and the analysis was performed with 20 replications for different values of K ( K = 2–11). The alignment charts were produced based on the STRUCTURE results acquired through the utilization of the CLUMPAK software (Kopelman et al., 2015). The ideal cluster value, denoted as K , was determined by analyzing the data with the STRUCTURE HARVESTER program. The approach utilized to ascertain this quantity was founded on the equation ( $\Delta K=m|L^{\prime \prime }(K)|/s[L(K)]$ ), as documented by Evanno et al. (2005).

**Table 2:** Thermal cycler conditions by touchdown PCR method.

Multiplex	Initial	Denaturation	Annealing	Extension	Total	Final
groups	denaturation				cycle	extension
M1	95 ${}^{\circ }$ C	95 ${}^{\circ }$ C	60–50 ${}^{\circ }$ C	72 ${}^{\circ }$ C	30	72 ${}^{\circ }$ C
	(5 min)	(40 s)	(40 s)	(1 min)		(10 min)
M2	95 ${}^{\circ }$ C	95 ${}^{\circ }$ C	60–50 ${}^{\circ }$ C	72 ${}^{\circ }$ C	30	72 ${}^{\circ }$ C
	(5 min)	(40 s)	(40 s)	(1 min)		(10 min)

## 3 Results

In this study, a total of 522 alleles were identified across 20 microsatellite loci. Table 3 displays the statistics of molecular genetic polymorphism.

The locus INRA0023 exhibited the greatest number of alleles (48), while the locus DRBP1 had the highest effective allele number (16.27). The loci BM1818 and DRBP1 had the highest polymorphic information content value (0.94). The anticipated level of heterozygosity (He) varied between 0.85 (ILSTS011) and 0.94 (BM1818, SRCRSP15, and DRBP1). The mean values of Wright's F statistics, namely $F_{\mathrm{IS}}$ , $F_{\mathrm{IT}}$ , and $F_{\mathrm{ST}}$ , were 0.111, 0.153, and 0.047, correspondingly. The mean values for $D_{\mathrm{ST}}$ , $H_{T}$ , and $G_{\mathrm{ST}}$ were calculated to be 0.026, 0.029 and 0.903, respectively. The data gathered from the 20 microsatellite loci utilized in the research were assessed for conformity with the Hardy–Weinberg equilibrium (HWE) through the application of the $\chi^{2}$ test. The analysis revealed that the distribution of alleles for all microsatellite markers examined did not adhere to the principles of the Hardy–Weinberg equilibrium. All null allele frequencies obtained from the microsatellites studied were found to be below 20 %. Table 4 presents genetic diversity statistics for Hair goat populations raised in various provinces.

**Figure 2:**
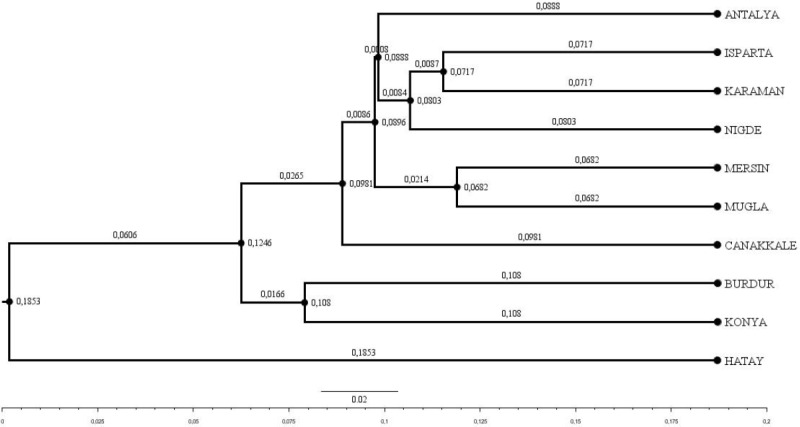
Dendrogram obtained using Nei's Da distance matrix (Nei, 1983) (bootstrap resampling methodology (1000 iterations)).

The Hair goat populations raised in Konya and Hatay exhibited the lowest (10.40) and highest (16.25) allele numbers, respectively. The $F_{\mathrm{IS}}$ (fixation index) values hold significant importance in defining population structures and determining the extent of heterozygosity losses. $F_{\mathrm{IS}}$ values ranged from 0.031 in Muǧla to 0.226 in Burdur. A total of 107 unique alleles were identified in Hair goat populations bred across 10 different provinces. However, only 25 alleles had a frequency above 5 %. The dendrogram depicting the genetic distance, as obtained from the study, is presented in Fig. 2.

**Table 3:** Polymorphism statistics of microsatellite loci studied.

Loci	Na	Ne	PIC	Ho	He	$F_{\mathrm{IS}}^{\#}$	$F_{\mathrm{IT}}^{\#}$	$F_{\mathrm{ST}}^{\#}$	$D_{\mathrm{ST}}$	$G_{\mathrm{ST}}$	$H_{T}$	HWE		F (Null)
												P	Sign.	
OarAE54	26	13.97	0.92	0.71	0.93	0.188	0.237	0.061	0.036	0.039	0.922	0.0000	${}^{***}$	0.1327
BM1818	36	16.11	0.94	0.90	0.94	0.018	0.056	0.038	0.011	0.012	0.943	0.0000	${}^{***}$	0.0189
OarFCB20	20	11.48	0.91	0.88	0.91	0.004	0.044	0.041	0.022	0.024	0.913	0.0000	${}^{***}$	0.0154
INRA0005	18	9.43	0.89	0.82	0.89	0.047	0.096	0.052	0.032	0.036	0.892	0.0000	${}^{***}$	0.0437
INRA0023	48	14.36	0.93	0.76	0.93	0.141	0.186	0.052	0.032	0.035	0.924	0.0000	${}^{***}$	0.1043
ILSTS011	30	6.48	0.83	0.58	0.85	0.272	0.328	0.076	0.047	0.057	0.83	0.0000	${}^{***}$	0.1911
SRCRSP9	24	9.31	0.88	0.73	0.89	0.138	0.177	0.045	0.026	0.029	0.89	0.0000	${}^{***}$	0.0999
SRCRSP15	27	15.60	0.93	0.84	0.94	0.084	0.120	0.039	0.018	0.019	0.938	0.0000	${}^{***}$	0.0539
TCRVB6	17	7.62	0.86	0.78	0.87	0.078	0.099	0.023	0.007	0.008	0.864	0.0000	${}^{***}$	0.0496
SRCRSP3	27	12.89	0.92	0.86	0.92	0.017	0.072	0.055	0.035	0.038	0.926	0.0036	${}^{**}$	0.0370
BM1329	27	10.02	0.89	0.67	0.90	0.230	0.252	0.028	0.007	0.008	0.898	0.0000	${}^{***}$	0.1473
McM0527	27	8.75	0.88	0.74	0.89	0.106	0.167	0.069	0.047	0.052	0.892	0.0000	${}^{***}$	0.0941
CSRD0247	29	9.05	0.88	0.64	0.89	0.230	0.272	0.054	0.031	0.035	0.88	0.0000	${}^{***}$	0.1692
INRABERN185	25	8.11	0.87	0.88	0.88	- 0.052	- 0.001	0.049	0.028	0.033	0.874	0.0000	${}^{***}$	- 0.0072
SRCRSP0023	27	16.06	0.93	0.76	0.94	0.119	0.164	0.050	0.031	0.033	0.938	0.0000	${}^{***}$	0.1050
ILSTS0087	16	7.87	0.86	0.75	0.87	0.100	0.142	0.046	0.025	0.029	0.876	0.0000	${}^{***}$	0.0772
SRCRSP0005	26	9.83	0.89	0.64	0.90	0.271	0.294	0.032	0.012	0.013	0.897	0.0000	${}^{***}$	0.1703
DRBP1	27	16.27	0.94	0.84	0.94	0.108	0.162	0.061	0.04	0.042	0.941	0.0000	${}^{***}$	0.0553
INRABERN172	20	9.59	0.89	0.85	0.90	0.012	0.053	0.041	0.023	0.025	0.891	0.0094	${}^{**}$	0.0258
HSC	25	15.12	0.93	0.79	0.93	0.130	0.154	0.027	0.007	0.008	0.934	0.0000	${}^{***}$	0.0870
Mean	26.10	11.40	0.87	0.77	0.90	0.111	0.153	0.047	0.026	0.029	0.903			

Na is the number of alleles, Ne is the number of effective alleles, PIC is the polymorphic information content, Ho is the observed heterozygosity, He is the expected heterozygosity, ${}^{\#}$ refers to Wright's F statistics (Weir and Cockerham, 1984), $D_{\mathrm{ST}}$ refers to interracial genetic diversity, $G_{\mathrm{ST}}$ refers to the coefficient of gene variation, HT refers to Nei's gene diversity, and HWE is the Hardy–Weinberg equilibrium; ${}^{*}$ indicates P<0.05 , ${}^{**}$ indicates P<0.01 , ${}^{***}$ indicates P<0.001 , and F (Null) indicates null allele frequency.

**Table 4:** Polymorphism statistics in Hair goat populations raised in different provinces.

Breeds	MNA	Mean heterozygosity		$F_{\mathrm{IS}}$	HWE	NPA		
		Ho	He			Freq.	Freq.	Total
						≥5 %	<5 %	
Antalya	14.85	0.80	0.87	0.107	7	1	4	5
Burdur	14.05	0.66	0.83	0.226	14	–	7	7
Çanakkale	15.90	0.80	0.88	0.103	8	–	12	12
Hatay	16.25	0.83	0.88	0.065	9	23	39	62
Isparta	13.75	0.72	0.85	0.172	7	–	3	3
Karaman	13.35	0.74	0.86	0.155	6	–	2	2
Konya	10.40	0.73	0.83	0.156	4	–	1	1
Mersin	14.80	0.78	0.86	0.111	11	1	1	2
Muǧla	15.10	0.85	0.87	0.031	6	–	7	7
Niǧde	14.75	0.72	0.86	0.179	7	–	6	6

MNA is mean number of alleles, Ho is observed heterozygosity, He is expected heterozygosity, $F_{\mathrm{IS}}$ is heterozygote deficiency, HWE is the number of loci not in the Hardy–Weinberg equilibrium ( P<0.05 ), and NPA is private alleles within-breed.

**Figure 3:**
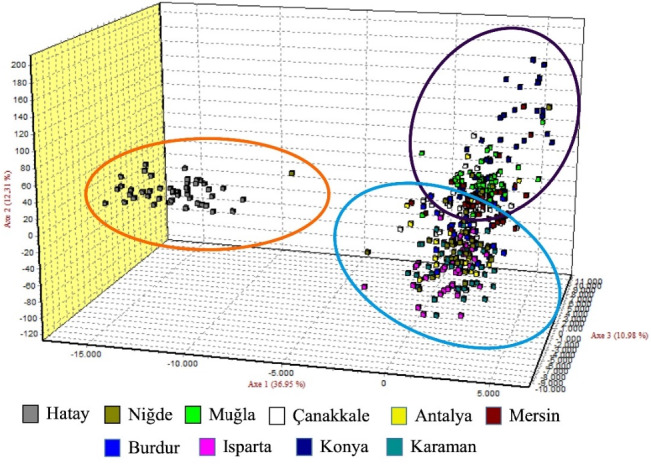
Factorial correspondence analysis (FCA) graph belonging to Hair goat populations raised in different provinces.

The graph illustrating the factorial correspondence analysis (FCA) is presented in Fig. 3. The graph provided by the FCA illustrates that the population of Hair goats raised in Hatay is distinct from those in other provinces, which appear to be more interconnected.

**Figure 4:**
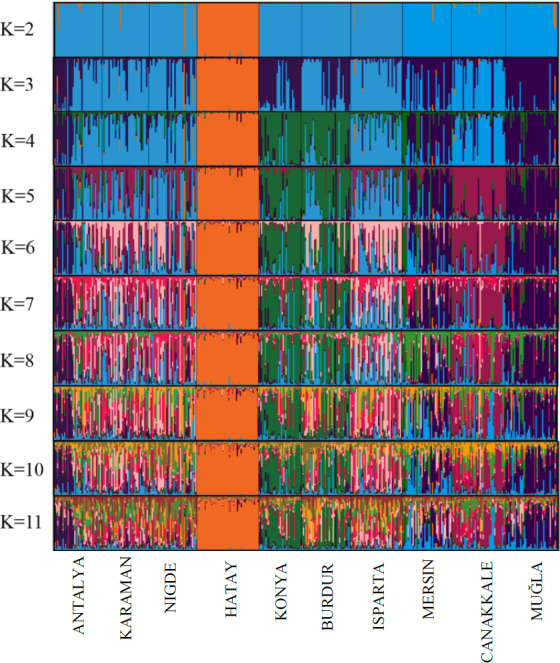
CLUMPAK plot of STRUCTURE assignment results ( K=2 –11).

The results of the STRUCTURE analysis, which involved varying clustering numbers ( K=2 –11), are depicted in Fig. 4. Additionally, Table 5 presents the findings, which include the estimation of posterior probabilities ([Ln Pr ( X|K )]) for different clustering numbers ( K ) and ΔK values.

## 4 Discussion

Hair goats have been raised in the mountainous regions of Türkiye for centuries. These goats have been an important source of income and nutrition for local people due to their ability to survive, especially in mountainous and difficult terrain (Daskiran et al., 2018). Although there are molecular genetic studies on the hair goat, there are a very limited number of genetic diversity studies with such a large sample. The study carried out in this context has revealed very important information about the population structures and intra-breed genetic diversity of Hair goats raised in different geographies of Türkiye.

**Table 5:** Estimation of posterior probabilities ([Ln Pr( X|K )]) for different clustering ( K ) and ΔK values.

K	[Ln Pr( X|K )]	ΔK
2	- 31729.285	–
3	- 31365.720	28.9092
4	- 31163.280	0.0962
5	- 30954.395	9.2563
6	- 30884.150	0.2319
7	- 30820.570	0.9537
8	- 30836.760	0.4794
9	- 30894.650	0.9807
10	- 30880.815	1.1579
11	- 30960.305	–

The allele numbers and average polymorphic information content obtained indicate that the 20 microsatellite loci utilized in the study exhibit a remarkably high degree of polymorphism. The high rates of observed heterozygosity, along with the average number of alleles detected at each locus, serve as significant indicators of the considerable genetic diversity present in the Hair goat populations under investigation.

Brito et al. (2017) reported Ho and He values of 0.374 and 0.369, respectively, in their genome-wide association study conducted on goats. Zaman and Shekar (2015) reported a total of 400 alleles in their investigation of Hair goats, whereas the current study identified 522 alleles. The results of this study on Hair goat populations indicate a higher number of alleles and molecular genetic parameter values, including polymorphic information content (PIC) and observed (Ho) and expected heterozygosity (He), compared to previous research on the topic. It is reported that there is a wide phenotypic variation within and between populations in Hair goat populations, which are bred in almost all regions of Türkiye and constitute a large part of the goat population (Cam et al., 2010; Elmaz and Saatcı, 2017). The findings obtained in the presented study, which was carried out with a large sample, revealed that Hair goat populations have a high level of genetic diversity and that the microsatellites used can be used successfully to identify the genetic diversity in Hair goat populations.

The levels of observed heterozygosity in this study were lower than those found in various other breeds bred in Türkiye (Bulut et al., 2016; Gumus, 2018; Gul et al., 2020; Karsli et al., 2022) but higher than those reported for certain other breeds (Agaoglu and Ertugrul, 2012; Gurler and Bozkaya, 2013; Gumus, 2018; Gul et al., 2020; Karsli et al., 2020; Tefiel et al., 2020). In terms of expected heterozygosity, a higher value has been obtained in this study compared to all previous studies (Agaoglu and Ertugrul, 2012; Gurler and Bozkaya, 2013; Bulut et al., 2016; Gumus, 2018; Gul et al., 2020; Tefiel et al., 2020; Karsli et al., 2020, 2022) conducted in Türkiye. The differences in the values revealed for some molecular genetic parameters in this study can be associated with the number of locations where the study was conducted and the sample size.

Upon examination of the $F_{\mathrm{IS}}$ values based on the microsatellites, it is notable that there is a reduction in heterozygosity at the INRABERN185 locus, which is one of the loci that was analyzed. The literature on the subject presents comparable results, as reported by Gurler and Bozkaya (2013) and Zaman and Shekar (2015). Hartl et al. (1997) reported, based on the $F_{\mathrm{ST}}$ classification of the loci studied, that the mean $F_{\mathrm{ST}}$ value (0.047) indicates relatively low genetic diversity among populations. The calculated $F_{\mathrm{ST}}$ value was lower than that reported in previous studies (Agaoglu and Ertugrul, 2012; Parejo et al., 2015; Bosman et al., 2015; Dominguez et al., 2018).

The mean coefficient of gene variation ( $G_{\mathrm{ST}}$ ) obtained in the study indicates that around 97.10 % of the identified genetic variation can be attributed to differences among individuals. However, the overall mean of $D_{\mathrm{ST}}$ indicates that there is not a significant level of inter-population variation. This finding, in fact, corroborates the previously mentioned results of $F_{\mathrm{ST}}$ and $G_{\mathrm{ST}}$ .

The $\chi^{2}$ test results indicate that the allele distributions of the 20 microsatellite markers examined are not in accordance with the Hardy–Weinberg equilibrium. Similar findings have been revealed in studies conducted on goats (Guang-Xin et al., 2019; Tefiel et al., 2020; Gul et al., 2020). It has been reported that deviations from the Hardy–Weinberg equilibrium (HWE) may occur due to artificial selection, and inbreeding may also contribute to these deviations (Granevitze et al., 2007; Guang-Xin et al., 2018). Due to the breeding programs implemented in all populations included in the study, controlled mating programs are carried out, and intensive selection is conducted. Considering this situation, it is thought that the resulting deviation from the Hardy–Weinberg equilibrium (HWE) is most likely due to selection.

The frequencies of null alleles for the 20 microsatellite loci utilized in this study were found to be below 20 %. This discovery provides tangible evidence that these 20 microsatellites can be utilized with confidence to delineate genetic variability. The MNa values obtained from the study's population were found to be higher than those reported in previous studies conducted on both domestic and foreign breeds (Zaman and Shekar, 2015; Awobajo et al., 2015; Al-Atiyat et al., 2015; Bulut et al., 2016; Tefiel et al., 2020).

Upon examination of the $F_{\mathrm{IS}}$ values, which represent the inbreeding coefficient with respect to populations, it was found that there was no loss of heterozygosity within the populations under study, with the exception of INRABERN185.

Although the Hair goat population studied had a total of 107 unique alleles, only 25 of them were found to have a frequency higher than 5 %. The Hatay Hair goat population exhibited the greatest number of private alleles. After analysis, it was found that the private alleles identified in the research were not particularly effective in distinguishing between populations, with the exception of those found in the Hair goat population specifically bred in the Hatay Province.

Upon examination of the dendrogram, it becomes apparent that three distinct clusters emerge. It is worth noting that the Hatay Hair goat population exhibits significant differences from other Hair goat populations. The factorial relationship analysis yielded an FCA graph that showed comparable results.

The outcomes derived from the STRUCTURE analysis were consistent with those of the dendrogram and FCA plot, as anticipated. In the STRUCTURE analysis, a noteworthy discovery is that the Hair goat population bred in the Hatay Province exhibits significant differentiation from other populations. In previous studies conducted in Hatay (Keskin and Bicer, 1997; Gul et al., 2020), it was reported that breeders had been crossbreeding Shami and Hair goat breeds in a non-systematic manner. This study concludes that the variation in Hair goats raised in Hatay, as compared to those raised in other locations, is a result of non-systematic crossbreeding. The most suitable number of groups was determined to be three based on the ΔK value obtained using the method described by Evanno et al. (2005). Considering the fact that all of the sampling was carried out from Hair goat populations, the results of the population structure analysis draw attention to the presence of significant admixtures in these populations. In Türkiye, goat breeding is carried out under extensive conditions, and it is even practiced as nomadic animal husbandry in some regions. It is believed that this admixture in populations may have arisen due to extensive breeding practices and nomadic animal husbandry. On the other hand, when considering the breeding locations of hair goats, it is noteworthy that, in addition to hair goats, Honamlı breeds are raised in Burdur Province, and Saanen breeds are also raised in Çanakkale Province. In the realm of goat breeds, the Honamlı breed distinguishes itself for its meat production capabilities, while the Saanen breed excels in terms of milk yield performance. Among these goat breeds, the Damascus and Saanen breeds have high milk yield performances. Therefore, the admixture observed in these regions can be associated with unsystematic crossbreeding practices carried out by breeders in order to improve specific yield traits. Upon examining the CLUMPAK graph, it is evident that there is a significant level of migration between populations, with the exception of the Hair goat populations raised in Hatay. The fact that Hatay Province has a completely different profile from other Hair goat breeding regions has revealed a very interesting situation. The Damascus breed, which stands out for its milk yield performance, is widely raised in Hatay Province. The difference in the population profile of hair goat populations in Hatay Province strengthens the suspicion that breeders use this breed for crossbreeding to enhance the milk yield of hair goats.

## 5 Conclusions

There is a limited amount of research on the molecular diversity of Hair goat populations in Türkiye. Upon considering other goat breeds in Türkiye, it is evident that the Hair goat is the most prevalent and dominant breed. This breed is known for its adaptability to various geographical locations. The current investigation has disclosed significant insights into the genetic variability of Hair goat populations reared across diverse regions of Türkiye, as well as the interrelationship among these populations. The microsatellites utilized in this study possess the potential to provide a precise depiction of the genetic variability present in the examined breeds. The findings of this investigation demonstrate that the microsatellite markers employed for the Hair goat populations exhibited polymorphism. Moreover, the populations under scrutiny exhibited a substantial level of genetic diversity, indicating the reliability of the markers used.

## Data Availability

The data underlying the findings of this research article are accessible from the corresponding author upon reasonable request.
